# Changes in Bone Turnover Markers after Osteoporotic Vertebral Compression Fractures in Males and Females

**DOI:** 10.1155/2022/5381601

**Published:** 2022-11-23

**Authors:** Zhongxiong Jia, Min Tang, Xiaojun Zhang, Wei Jiang, Jieliang Shen, Nian Zhou, Jie Hao

**Affiliations:** ^1^Department of Orthopedics, The Second People's Hospital of Yibin, Yibin, Sichuan, China 644000; ^2^Department of Oncology, The First Affiliated Hospital of Chongqing Medical University, Chongqing, China 400016; ^3^Department of Orthopedics, The First Affiliated Hospital of Chongqing Medical University, Chongqing, China 400016

## Abstract

**Background:**

To explore the normal changes in bone turnover markers (BTMs) and the correlations between the different BTMs after osteoporotic vertebral compression fracture (OVCF). Meanwhile, we explored the related differences that exist between sexes.

**Methods:**

A total of 130 OVCF patients were retrospectively reviewed. Using IBM SPSS 19.0 statistical software, the differences in the levels of BTMs and clinical parameters between sexes were assessed using Student's unpaired *t* test, and one-way ANOVA was used for the comparison of the three groups of samples. The correlations between P1NP, CTX, and clinical factors were assessed using Pearson's correlation coefficient.

**Results:**

P1NP was 52.15 ng/ml within two weeks in male patients, and the level increased to 96.33 ng/ml after 12 weeks; in female patients, the increase was not as obvious as in male patients. CTX in male patients reached as much as approximately twice the initial value after 12 weeks. However, the situation in female patients was diverse. CTX was 0.58 ng/ml within two weeks and increased to 0.61 ng/ml within 2-12 weeks after the onset of OVCF. Subsequently, CTX decreased suddenly after 12 weeks. The increase in P1NP levels within 2 weeks after OVCF was significantly correlated with the levels of osteocalcin (OC) and bone-specific alkaline phosphatase (BAP). Changes in CTX within 2 weeks after OVCF were considerably related to phosphorus, 25 hydroxyvitamin D (25-OHD), OC, and BAP.

**Conclusion:**

The levels of P1NP and CTX increased differently in males and females after OVCF. The levels of OC and BAP were correlated with the levels of P1NP and CTX within 2 weeks of OVCF.

## 1. Introduction

Osteoporosis, as a common metabolic bone disease, is characterized by decreased bone mineral density (BMD) and an increased risk of bone fracture. Over the last three decades, the occurrence of this disease has increased by nearly 300% in China and other developing countries [1] and has become a major cause of vertebral compression fractures in aged people [2]. According to the statistics of the China Aging Research Center, there were 202 million elderly people in China [3] and many of them have suffered compression fractures of the vertebral body due to osteoporosis [4].

Conventionally, bone is a dynamic organ undergoing constant anabolism and catabolism, and there is a balance between bone formation and bone resorption. During bone resorption, osteoclasts remove small amounts of bone from the different parts of the bone, which are then excreted in the blood or urine [5]. The amount of bone components or their catabolites in blood and urine (i.e., substances such as the CTX) may reflect bone resorption activity. Then, osteoblasts arrive at the newly formed pit and begin bone formation. Blood concentrations of molecules such as OC and BAP that are released by osteoblasts can reflect bone-forming activity [5]. Parathyroid hormone (PTH) plays a key role in Ca metabolism because it regulates renal production of 1,25(OH)_2_D [6], which is released from the kidney and is the most important regulator of increased intestinal Ca absorption [6]. Bone remodeling is a dynamic process of spatiotemporal coupling that involves bone resorption mediated by osteoclasts and bone formation induced by osteoblasts [7]. However, under certain pathological conditions, such as osteoporosis or Paget's disease, bone metabolism is usually changed [8, 9].

Studies conducted over the last decade have examined the various potential candidate markers of bone remodeling and their variability [10]. BTMs are commonly used to assess bone turnover and can be divided into two categories: markers of bone formation and markers of bone absorption. In recent years, P1NP and CTX have been recommended as the BTM of choice in predicting fracture risk and monitoring antiresorptive treatment in osteoporosis [11] and have been used in clinical studies regarding osteoporosis. On the one hand, CTX is defined as the bone resorption marker. However, it cannot be ignored that a series of factors might have a critical influence on its concentration, such as the circadian rhythm [12, 13], renal insufficiency [14], age [15], food intake [12, 16], and exercise [17]. On the other hand, P1NP produced by the formation of type I collagen, a major component of bone matrix, by amino-terminal and carboxy-terminal splicing of type I procollagen in osteoblasts [18] is a sensitive bone formation marker that has a less pronounced circadian rhythm compared to CTX [19]. In addition, markers such as P1NP and OC are also affected by food and drink but to a lesser degree than CTX, while BAP is not affected. Furthermore, to the best of our knowledge, the relationship between BTMs and PTH and 25-OHD remains unclear [18, 20], although the latter has an important effect on the regulation of calcium and phosphorus metabolism and also plays a major role in osteoporosis.

Recently, there has been increasing interest in changes in BTMs after a fracture. Li et al. [21] revealed that after elderly hip fracture surgery, P1NP levels showed less than 4 times elevation and CTX and OC levels were less than 2-fold increased. However, the changes in the aforementioned BTMs and the correlations among the different BTMs after OVCF have received little attention. So, we conducted this study to explore the normal changes in BTMs and the correlations among the different BTMs after OVCF. Meanwhile, we explored the differences that exist between males and females.

## 2. Materials and Methodology

### 2.1. Ethics Statement

This retrospective study was carried out in accordance with the guidelines of the Declaration of Helsinki and its amendments. The research protocol for the study was approved by the Ethical and Scientific Committees of The First Affiliated Hospital of Chongqing Medical University (Chongqing, China).

### 2.2. Patients

A total of 130 OVCF patients who were treated at The First Affiliated Hospital of Chongqing Medical University from 2016 to June 2017 were retrospectively selected to enroll in the study. Given the processes of fracture healing, i.e., the inflammation and hematoma phase, the formation of a cartilaginous callus phase, and the remodeling of the callus phase, patients were divided into the following 3 groups according to their chief complaints at admission: group 1: the time from symptom onset to visit was less than 2 weeks; group 2: within 2 to 12 weeks after the onset of symptoms; and group 3: the time from symptom onset to medical treatment was more than 12 weeks. All included patients met the following inclusion criteria: (1) patients diagnosed with OVCF and (2) based on the research factors, patients who had complete clinical information and laboratory data. The exclusion criteria were as follows: (1) vertebral fracture due to violence; (2) prior history of antiosteoporosis therapy or receiving drugs with potential effects on bone metabolism; (3) secondary causes of osteoporosis; (4) diabetes mellitus; and (5) clinical evidence of tumors.

### 2.3. Diagnosis and Treatments

Patients underwent a series of imaging studies, including dual-energy X-ray, which is widely considered the standard of reference in assessing BMD and fracture risk [22]; magnetic resonance imaging (MRI) of the spine; and venous blood sampling in the fasting state to complete the relevant tests, which provided data on serum bone metabolism indicators, etc., and were conducted within one week and one day after admission. The diagnostic criteria of OVCF were as follows: (1) the injury model of the patient was low-energy injury or no history of injury; (2) MRI showed a low signal change of injured vertebrae; T2 image showed high signal change; and (3) dual-energy X-ray examination showed that the *T* value of BMD was less than -2.5. After exclusion of contraindications, percutaneous vertebroplasty and percutaneous kyphoplasty were performed according to the specific conditions of the patients.

### 2.4. Clinical Data Collection

The clinicopathological characteristics of the OVCF patients, including sex, age, body mass index (BMI), and biochemical indices, such as P1NP, CTX, 25-OHD, OC, BAP, lumbar spine BMD *T*-score, and hip BMD *T*-score measured by dual X-ray absorptiometry (DXA), PTH, calcium (Ca), and phosphorus concentrations, were retrospectively and systematically retrieved from the medical records. According to the World Health Organization definitions [23], patients with a BMD of 2.5 standard deviations (SD) lower (*T*-scores ≤ −2.5) than the peak BMD of the same sex were defined as having osteoporosis.

### 2.5. Statistical Analysis

Using IBM SPSS 19.0 statistical software, the measurement data are presented as the mean and SD, and nonnormally distributed data are expressed as the median (25% and 75%). Differences in the levels of BTMs and clinical parameters between sexes were assessed using Student's unpaired *t* test, and one-way ANOVA was used for the comparison of the three groups of samples. The correlations between P1NP and CTX and clinical factors were assessed using Pearson's correlation coefficient. A *P* value < 0.05 indicated that the difference was statistically significant.

## 3. Results

### 3.1. Clinical Characteristics of Patients

**Figure 1:**
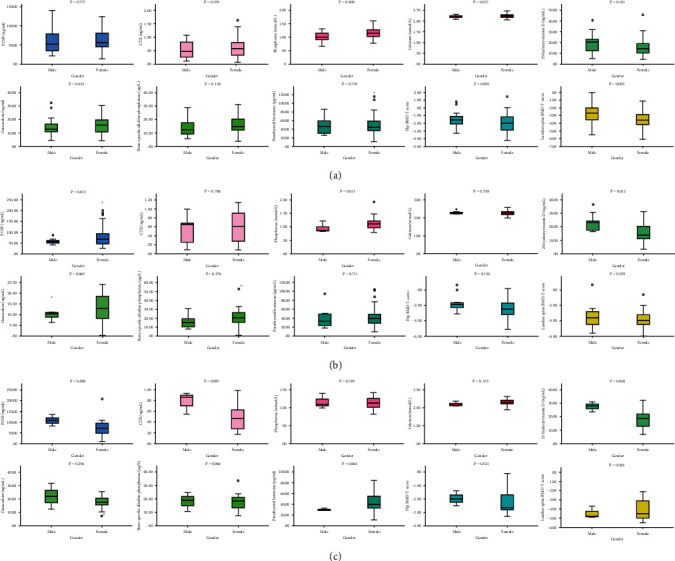
General characteristics of biochemistry parameters between male and female OVCF patients.

The clinical characteristics of the 130 patients, including 32 (24.6%) males and 98 (75.4%) females, are presented in Table 1. The average ages of males and females were 72.59 ± 9.80 y and 71.66 ± 9.62 y, respectively. The mean 25-OHD (*P* = 0.002), lumbar spine BMD *T*-score (*P* = 0.006), and hip BMD *T*-score (*P* = 0.012) in male patients were significantly higher than those in females, while the mean phosphorus (*P* = 0.001) value in male patients was significantly lower than that in female patients. Regarding the indices of age (*P* = 0.637), BMI (*P* = 0.412), P1NP (*P* = 0.314), CTX (*P* = 0.684), PTH (*P* = 0.556), OC (*P* = 0.367), Ca (*P* = 0.094), and BAP (*P* = 0.055), there were no significant differences between sexes (Table 1). The basic biochemistry parameter levels between male and female OVCF patients are shown in Figure 1. Within two weeks, most of the parameters between the two cohorts, such as phosphorus (*P* = 0.008), Ca (*P* = 0.037), and lumbar spine BMD *T*-score (*P* = 0.005), were significantly different. Within 12 weeks, the difference levels of indicators between sexes were statistically significant, e.g., phosphorus (*P* = 0.013), P1NP (*P* = 0.013), and 25-OHD (*P* = 0.012). Over 12 weeks, the various values of 25-OHD between the two subgroups were significantly different (*P* = 0.040).

### 3.2. The Time Course of P1NP Level

**Figure 2:**
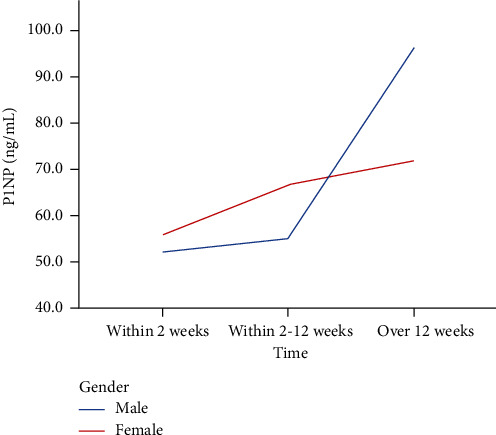
Change in P1NP during the OVCF healing process.

P1NP was significantly increased in both male and female patients after OVCF. P1NP concentration was 52.15 ng/ml within two weeks in male patients and increased to 96.33 ng/ml after 12 weeks, suggesting that P1NP in male patients can reach approximately twice the initial value 12 weeks after fracture occurrence, but the increase in female patients was not as obvious as in male patients. The changes in P1NP levels during the healing process of OVCFs are shown in Table 2 and Figure 2. Analysis of P1NP levels at different times in male and female patients was carried out. In male patients, compared to P1NP levels after 12 weeks, P1NP levels within 2 weeks and within 2-12 weeks after OVCF were significantly different (*P* = 0.026 and *P* = 0.044, respectively). However, there was no significant difference between P1NP levels within 2-12 weeks and P1NP levels within 12 weeks (*P* = 0.897). Regarding the female patients, there were no significant differences between P1NP levels within 2 weeks and P1NP levels within 2-12 weeks (*P* = 0.163) and P1NP levels after 12 weeks (*P* = 0.627). Moreover, compared to P1NP levels over 12 weeks, P1NP levels within 12 weeks were not significant (*P* = 0.602).

### 3.3. The Time Course of CTX Level

**Figure 3:**
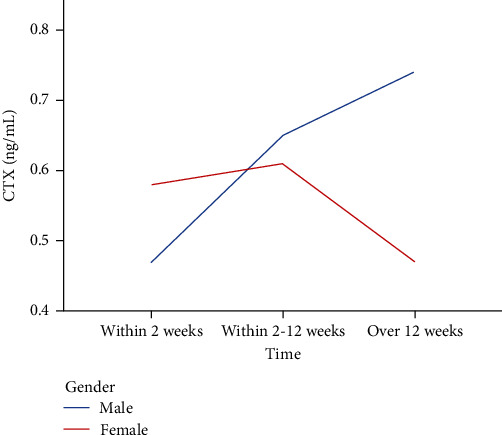
Change in CTX during the healing process of OVCF.

CTX was sustainably increased in male patients with OVCF. CTX was 0.47 ng/ml within two weeks and reached a higher value over 12 weeks than within two weeks. The changes in CTX levels during the healing process of OVCF are shown in Table 3 and Figure 3. It is suggested that CTX concentrations in male patients can reach 0.74 ng/ml, approximately twice the initial value, after 12 weeks, but there was no statistical significance (*P* = 0.185). However, the situation in female patients was diverse. CTX concentration was 0.58 ng/ml within two weeks and increased to 0.61 ng/ml within 2-12 weeks after the onset of OVCF. Thereafter, CTX concentration decreased suddenly after 12 weeks. There was no significant difference (*P* = 0.798).

### 3.4. P1NP and Clinical Factors

**Figure 4:**
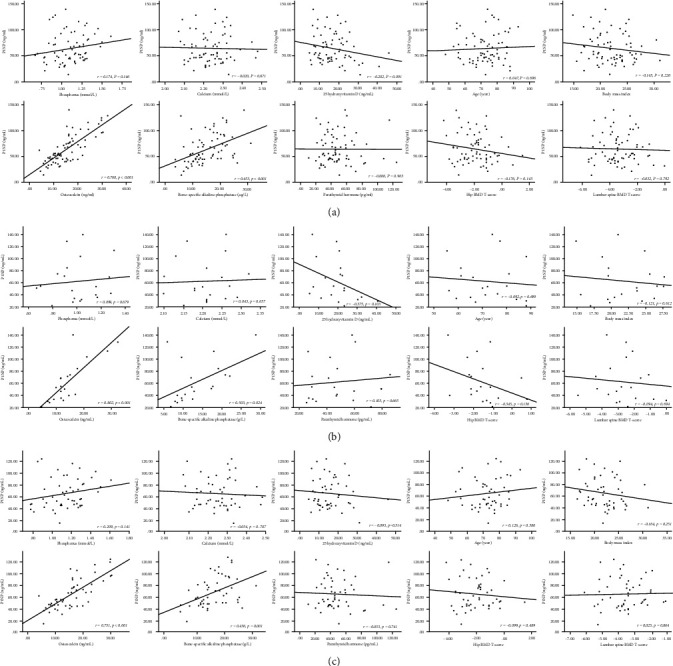
Scatter diagrams of P1NP level and age, phosphorus, Ca, 25-OHD, BMI, PTH, hip BMD *T*-score, lumbar spine BMD *T*-score, OC, and BAP values during the first 2 weeks of OVCF healing. (a) Total; (b) males; (c) females.

In general, correlation analysis showed that the increase in P1NP levels within 2 weeks after OVCF was significantly correlated with levels of OC (*r* = 0.760, *P* < 0.001) and BAP (*r* = 0.453, *P* < 0.001), while there was no statistical correlation between age (*P* = 0.696), phosphorus (*P* = 0.146), Ca (*P* = 0.871), 25-OHD (*P* = 0.091), BMI (*P* = 0.226), PTH (*P* = 0.963), hip BMD *T*-score (*P* = 0.143), and lumbar spine BMD *T*-score (*P* = 0.792). Scatter diagrams of P1NP and clinical factors during the first two weeks of OVCF healing are depicted in Figure 4. With regard to male patients, the level of P1NP within 2 weeks was associated with the level of OC (*r* = 0.802, *P* < 0.001) and BAP (*r* = 0.503, *P* = 0.024). Regarding the women, the value of P1NP within 2 weeks was also related to levels of OC (*r* = 0.751, *P* < 0.001) and BAP (*r* = 0.436, *P* = 0.001).

### 3.5. CTX and Clinical Factors

**Figure 5:**
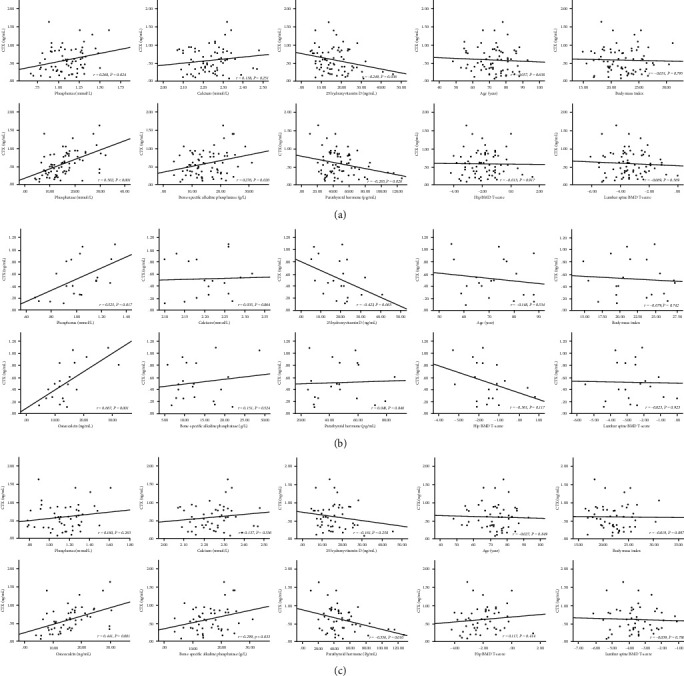
Scatter diagrams of CTX level and age, phosphorus, Ca, 25-OHD, BMI, PTH, hip BMD *T*-score, lumbar spine BMD *T*-score, OC, and BAP values during the first 2 weeks of OVCF healing. (a) Total; (b) males; (c) females.

Scatter diagrams of CTX concentrations and clinical parameters during the first two weeks of OVCF healing are presented in Figure 5. Overall, CTX within 2 weeks after OVCF was considerably related to phosphorus (*r* = 0.268, *P* = 0.024), 25-OHD (*r* = 0.249, *P* = 0.036), OC (*r* = 0.502, *P* < 0.001), and BAP (*r* = 0.276, *P* = 0.020), while there was no statistical correlation between age (*P* = 0.638), Ca (*P* = 0.251), BMI (*P* = 0.795), PTH (*P* = 0.963), hip BMD *T*-score (*P* = 0.917), and lumbar spine BMD *T*-score (*P* = 0.569). In male OVCF patients, the level of CTX within 2 weeks was associated with phosphorus (*r* = 0.525, *P* = 0.017) and BAP (*r* = 0.667, *P* = 0.001). Regarding the female patients, the value of CTX was significantly related to OC (*r* = 0.441, *P* = 0.001), PTH (*r* = 0.356, *P* = 0.010), and BAP (*r* = 0.299, *P* = 0.033).

## 4. Discussion

Regarding the early changes in bone-specific turnover markers and the correlations among the different BTMs after OVCF in female and male patients, which have never before been systematically and comprehensively analyzed, we carried out the present trial for further exploration.

Consistent with the previous studies, our findings suggest that male sex was positively associated with a high mean 25-OHD level (*P* = 0.002), lumbar spine BMD *T*-score (*P* = 0.006), and hip BMD *T*-score (*P* = 0.012). To understand what might contribute to the phenomenon, we further found that compared to bone tissue receptor activator of nuclear factor kB ligand (RANKL) expression in men's bone tissues, the bone tissues' RANKL expression in women was 20% higher, which suggested that higher expression of RANKL accounts for lower BMD in women [24]. This finding is in agreement with observations that men have higher bone mineral density [2, 25–28]. Indeed, the decline in estrogens in postmenopausal women is associated with a decrease in BMD, which indicates that lower estrogen levels influence bone mineral density [29]. Additionally, a growing number of studies have demonstrated that females are more likely to suffer vitamin D deficiency because the levels of vitamin D were higher in males in all age sets [30] compared to women, possibly reflecting higher cosmetic use, more indoor activity, or more coverage of skin outdoors among females [31]. It is acknowledged that 25-OHD has a vital influence on calcium absorption and bone health, and 25-OHD is essential for muscle performance, balance, and reduced risk of falling [32]. Thus, until now, several studies have recommended that vitamin D doses greater than 1,000 IU or even 4,000 IU per day are needed [33, 34]. According to the results of our study, the male vitamin D doses should be greater than females in the antiosteoporosis program.

With regard to P1NP, a sensitive bone formation marker. In the current trial, we detected that P1NP concentration was significantly increased in both male and female patients after OVCF. In keeping with our conclusions, there is good evidence that PINP increases during the early period after fracture [35, 36]. Unfortunately, among those studies, further research on the time course of PINP levels in female and male patients has not been carried out. We speculated that the mobilization of bone metabolism after fracture in men is stronger than in women and may account for P1NP levels being considerably increased in male patients, while the trend in female patients is not as obvious. However, randomized controlled trials (RCTs) and prospective trials with more participants are needed to confirm these results in the future. Concerning CTX, the level was sustainably increased in male patients with OVCF. However, the situation in female patients was different. CTX concentration was 0.58 ng/ml within two weeks and increased to 0.61 ng/ml within 2-12 weeks after the onset of OVCF. Thereafter, CTX decreased suddenly after 12 weeks. Ivaska et al. [37] conducted a prospective study of 113 elderly female patients and revealed that CTX increased during 2 weeks following fracture and decreased in the following 2–3 months. This conclusion is generally consistent with our results. We all have found that CTX will suffer a decreasing trend after a period of time, although the definitive cutoff time is unclear. Taken together, the time courses of P1NP and CTX changes showed a positive increasing tendency in male patients, while there was a relatively slow increasing trend and even a decreasing trend after some time in female patients. At the same time, PINP is considered a sensitive bone formation marker, and CTX is defined as the bone resorption indicator. The processes of anabolism and catabolism of bone are relatively more active in men. Based on the current evidence, the underlying mechanisms contributing to this situation remain unclear, and comprehensive research, which can facilitate these biomarkers being widely used in the clinic, is essential.

In general, the increase in P1NP levels within 2 weeks after OVCF was correlated with changes in OC and BAP. In particular, the level of P1NP within 2 weeks was associated with OC and BAP changes in male patients. Regarding the women, the value of P1NP within 2 weeks was related to OC and BAP levels as well. This is easily explained because PINP, OC, and BAP are all secreted by osteoblasts.

Similarly, in summary regarding CTX, the value of this indicator within 2 weeks after OVCF was considerably related to levels of phosphorus, 25-OHD, OC, and BAP, while there was no statistical correlation with age, Ca, BMI, PTH, hip BMD *T*-score, and lumbar spine BMD *T*-score. When we performed the analysis in male and female patients, our results revealed that the level of CTX was significantly associated with levels of phosphorus and BAP in male patients, while the level of CTX was related to OC, PTH, and BAP concentrations in female patients.

Our study has several limitations that need to be acknowledged. First, our study is a retrospective study conducted in a single institution, and there may be selection bias. Second, the sample size over 12 weeks was relatively small. Third, all the indices were assessed at a single time point only during the process and might be affected by various pathological and physiological conditions. Future work is needed to validate these preliminary findings and disclose the underlying mechanisms.

## 5. Conclusion

The levels of P1NP and CTX increased differently in males and females after OVCF. OC and BAP levels were correlated with P1NP and CTX concentrations within 2 weeks of OVCF. Females are more likely to be 25-OHD deficient than males.

## Figures and Tables

**Table 1:** The demographic and baseline characteristics of patients.

Characteristics	No. pts	Age (years)	BMI (kg/cm^2^)	25-OHD (ng/ml)	P1NP (ng/ml)	CTX (ng/ml)	OC (ng/ml)	BAP (*μ*g/L)	PTH (pg/ml)	Ca (mmol/L)	P (mmol/L)	Hip *T*-score	Lumbar *T*-score
Male	32	72.59 ± 9.80	21.46 ± 3.43	21.11 ± 8.38	65.12 ± 32.25	0.55 ± 0.30	14.00 ± 6.84	14.78 ± 6.58	43.78 ± 19.42	2.23 ± 0.08	1.00 ± 0.18	−1.61 ± 1.03	−3.05 ± 1.49
Female	98	71.66 ± 9.62	22.07 ± 3.69	16.17 ± 7.37	73.13 ± 40.85	0.58 ± 0.33	15.22 ± 6.53	17.78 ± 6.90	46.42 ± 22.68	2.26 ± 0.13	1.14 ± 0.20	−2.19 ± 1.16	−3.75 ± 1.12
*P* value		0.637	0.412	0.002	0.314	0.684	0.367	0.055	0.556	0.094	0.001	0.012	0.006

No. pts: number of patients; BMI: body mass index; 25-OHD: 25 hydroxyvitamin D; P1NP: N-propeptide of type 1 collagen; CTX: C-terminal crosslinking telopeptides of type 1 collagen; OC: osteocalcin; BAP: bone-specific alkaline phosphatase; PTH: parathyroid hormone; Ca: calcium; P: phosphorus.

**Table 2:** The time course of the P1NP level.

Gender	Parameter	Within 2 weeks	Within 2-12 weeks	Over 12 weeks
Total	No. pts	71	42	17
	ln P1NP	4.06 ± 0.45	4.21 ± 0.54	4.26 ± 0.66
	P1NP	55.21 (44.10, 81.67)	62.98 (44.73, 89.67)	82.13 (50.77, 100.80)

*P* value		0.411^a^	0.068^b^	0.253^c^

Male	No. pts	20	9	3
	ln P1NP	3.99 ± 0.54	4.02 ± 0.23	4.66 ± 0.25
	P1NP	52.15 (34.07, 81.71)	55.10 (45.92, 64.82)	96.33 (82.13, 117.84)

*P* value		0.897^a^	0.026^∗^^b^	0.044^∗^^c^

Female	No. pts	51	33	14
	ln P1NP	4.09 ± 0.42	4.26 ± 0.59	4.17 ± 0.69
	P1NP	55.94 (44.77, 81.67)	66.73 (44.22, 93.09)	71.88 (46.40, 95.60)

*P* value		0.163^a^	0.627^b^	0.602^c^

^∗^ *P* value statistically significant (*P* < 0.05). No. pts: number of patients; ln P1NP: find ln for P1NP; P1NP: N-propeptide of type 1 collagen. ^a^Comparison within 2 weeks and 2-12 weeks. ^b^Comparison within 2 weeks and after 12 weeks. ^c^Comparison between 2-12 weeks and over 12 weeks.

**Table 3:** The time course of the CTX level.

Gender	Parameter	Within 2 weeks	Within 2-12 weeks	Over 12 weeks
Total	No. pts	71	42	17
	InCTX	−0.75 ± 0.68	−0.80 ± 0.78	−0.75 ± 0.55
	CTX	0.54 (0.31, 0.82)	0.61 (0.24, 0.81)	0.52 (0.28, 0.84)

*P* value		0.372^a^	0.913^b^	0.102^c^

Male	No. pts	20	9	3
	InCTX	−0.86 ± 0.69	−0.83 ± 0.79	−0.27 ± 0.29
	CTX	0.47 (0.26, 0.83)	0.65 (0.24, 0.73)	0.74 (0.55, 0.89)

*P* value		0.897^a^	0.185^b^	0.246^c^

Female	No. pts	51	33	14
	InCTX	−0.70 ± 0.67	−0.79 ± 0.79	−0.85 ± 0.55
	CTX	0.58 (0.33, 0.82)	0.61 (0.25, 0.91)	0.47 (0.27, 0.68)

*P* value		0.563^a^	0.485^b^	0.798^c^

No. pts: number of patients; ln P1NP: find ln for P1NP; P1NP: N-propeptide of type 1 collagen. ^a^Comparison within 2 weeks and 2-12 weeks. ^b^Comparison within 2 weeks and after 12 weeks. ^c^Comparison between 2-12 weeks and over 12 weeks.

## Data Availability

The data used to support the findings of this study are included within the article.

## Abbreviations

BTMs: Bone turnover markers
OVCF: Osteoporosis vertebral compress fracture
P1NP: N-Propeptide of type 1 collagen
CTX: C-Terminal crosslinking telopeptides of type 1 collagen
OC: Osteocalcin
BAP: Bone-specific alkaline phosphatase
25-OHD: 25 hydroxyvitamin D
BMD: Bone mineral density
PTH: Parathyroid hormone
MRI: Magnetic resonance imaging
BMI: Body mass index
DXA: Dual X-ray absorptiometry
Ca: Calcium
SD: Standard deviation
RANKL: Receptor activator of nuclear factor *κ*B ligand.
